# Assessment of the Presence of Hepatitis E Virus in Surface Water and Drinking Water in Portugal

**DOI:** 10.3390/microorganisms8050761

**Published:** 2020-05-19

**Authors:** Daniel Salvador, Célia Neto, Maria João Benoliel, Maria Filomena Caeiro

**Affiliations:** 1 Instituto de Saúde Ambiental, Faculdade de Medicina da Universidade de Lisboa, Avenida Prof. Egas Moniz, Edifício Egas Moniz, Piso 0, Ala C, 1649-028 Lisboa, Portugal; daniel.salvador@adp.pt; 2 Direção de Laboratório e Controlo da Qualidade da Água (LAB) da Empresa Portuguesa das Águas Livres (EPAL), Avenida de Berlim, 15, 1800-031 Lisboa, Portugal; celianet@adp.pt (C.N.); mjbenoliel@adp.pt (M.J.B.); 3 Departamento de Biologia Vegetal, Faculdade de Ciências da Universidade de Lisboa, Centro de Estudos do Ambiente e do Mar (CESAM), Edifício C2—Piso 2, Campo Grande, 1749-016 Lisboa, Portugal

**Keywords:** drinking water, enteric viruses, Hepatitis E virus, RT-qPCR, surface water, Vero E6 cell line, viral infectivity, water quality, water treatment

## Abstract

Hepatitis E virus (HEV) is a non-enveloped single-stranded positive-sense RNA virus, belonging to the Hepeviridae family, resistant to environmental conditions, and transmitted by the consumption of contaminated water. This virus is responsible for both sporadic and epidemic outbreaks, leading to thousands of infections per year in several countries, and is thus considered an emerging disease in Europe and Asia. This study refers to a survey in Portugal during 2019, targeting the detection and eventual quantification of enteric viruses in samples from surface and drinking water. Samples positive for HEV RNA were recurrently found by reverse transcription quantitative PCR (RT-qPCR), in both types of matrix. The infectivity of these samples was evaluated in cultured Vero E6 cells and RNA from putative viruses produced in cultures evidencing cytopathic effects and was subjected to RT-qPCR targeting HEV genomic RNA. Our results evidenced the existence of samples positive either for HEV RNA (77.8% in surface water and 66.7% in drinking water) or for infectious HEV (23.0% in surface water and 27.7% in drinking water). These results highlight the need for effective virological control of water for human consumption and activities.

## 1. Introduction

Diseases transmitted by water and food have high socioeconomic impacts on public health all around the world, especially at a time when climate change has caused alterations in the occurrence, distribution and seasonal variation of several pathogens, increasing the risk of exposure [1,2,3]. Viruses are among the most worrying groups of pathogens. Enteric viruses, which include, among others, enteroviruses, noroviruses, rotaviruses, Hepatitis A virus and Hepatitis E virus (HEV) are excreted in large quantities in human feces and transmitted mainly by the fecal–oral route [3,4,5,6]. They do not have a lipid envelope and possess a robust protein capsid, which is responsible for their high resistance to environmental stress such as heat, extreme pH, desiccation, and organic solvents [2]. Typical treatments used to inactivate/remove bacterial pathogens or enveloped viruses (for example, *Influenza*) in food and water, such as filtration and chemical oxidants, may not be effective against this group of viruses. In Korea, infectious enteric viruses such as adenovirus and enterovirus were detected in tap water, despite treatments [7]. Further, their stability also makes them quite resistant to the most common sanitation treatments and allows their maintenance in wastewater for long periods [2,8,9]. Recently, in Canada, several infectious enteric viruses, such as rotavirus, have been detected in surface water in the country’s largest rivers, where many wastewater sources are discharged [10].

According to the World Health Organization (WHO), enteric viruses have moderate to high significance in human health [4] and the existence of 20 million infections worldwide was estimated for HEV alone, with 3.3 million symptomatic cases of acute hepatitis E. In 2015, HEV caused approximately 44,000 deaths worldwide, representing 3.3% of viral hepatitis mortality [11]. In Europe, 68,000 HEV infections had recently been estimated in France, 100,000 in the United Kingdom and 300,000 in Germany, per year. The number of infections has been increasing dramatically, and now hepatitis E is considered an emerging disease in Europe and Asia [2,5].

HEV is a non-enveloped single-stranded positive-sense RNA virus belonging to the Hepeviridae family [12,13]. HEV is transmitted mainly by the consumption of contaminated water or contaminated undercooked or raw food such as vegetables, meat (pork, mutton, rabbit, poultry) and dairy products [2,5,14,15]. It is important to note that contaminated water is not only a cause of direct transmission but is also related to indirect transmission due to its use in agriculture practices, namely in the irrigation of vegetables, where the virus may accumulate and be delivered as infectious particles [16,17,18,19,20]. Although less frequent, HEV can also be transmitted by transfusions with contaminated blood and by vertical (mother-to-child) transmission [14,21,22].

HEV was responsible for both sporadic and epidemic outbreaks in several countries [23,24], and has been detected in natural waters from Colombia, Italy, and Sweden [9,25,26], in drinking water in Sweden and India [9,27], and in wastewater in Italy, Sweden, Pakistan, Colombia, Portugal and Spain [14,25,28,29,30,31].

After entering the human body, HEV is associated with clinical manifestations such as jaundice, vomiting, loss of appetite, fatigue, fever, darkened urine, hepatalgia and hepatomegaly. Although less common, this virus may also be associated with neurological complications such as Guillain–Barré syndrome, neuralgic amyotrophy, inflammatory polyradiculopathy, ataxia/encephalitis, and peripheral neuropathy. The mortality rate associated with HEV is approximately 2% in the general population, although in pregnant women the rate increases to 20% [21,32,33]. HEV infectious dose is not known [3].

**Figure 1:**
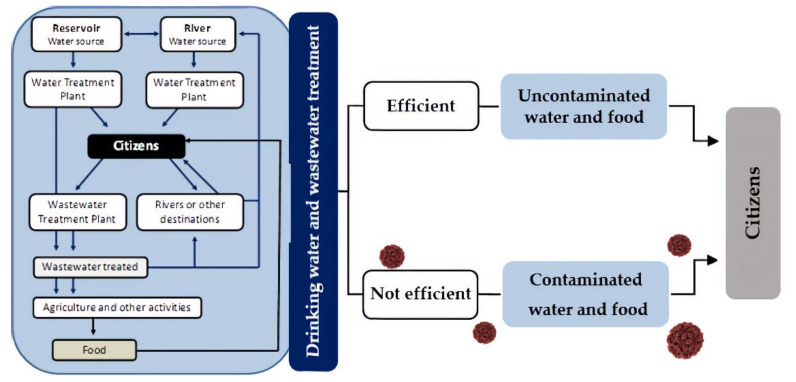
Schematic representation of water collection and distribution networks and their influence on human health.

A vaccine to prevent HEV infection was developed in China but is not yet available in most countries [11]. Antibiotics are ineffective and only a small number of antivirals, such as ribavirin have been indicated to treat infected individuals [34,35]. For all these reasons, there is a growing need for an effective surveillance system for the detection and quantification of this pathogen, mainly in water sources delivered to large populations, in order to reduce its transmission to humans and prevent potential epidemic situations with remarkable human and economic impacts (Figure 1) [3,5,36].

This study aimed to evaluate the presence of HEV in two sources of surface water and in drinking water sampled at two water treatment plants (WTPs), located at the central region of Portugal and at a point in the water distribution network. Our first approach was a quantitative molecular method (reverse transcription quantitative PCR (RT-qPCR)) applied to RNA extracted from sampled water in order to identify HEV-positive samples. A second approach, applied to HEV-positive samples, consisted of their inoculation in cultured cells (Vero E6 cell line); RNA was extracted from putative viral particles produced in these cells and HEV replication was detected/confirmed by RT-qPCR.

## 2. Materials and Methods 

### 2.1. Study Sites

This study was carried out in two water matrices (surface water and drinking water) localized at the central region of Portugal. The sampling points of surface water were in a river and in a dam reservoir. Two sampling points of drinking water were located at the end of the treatment process in two water treatment plants (WTPs) and another one, at one point in the water distribution network. WTP_R treats surface water from the river and WTP_D treats surface water from the dam reservoir. In WTP_R, surface water goes through the following treatment processes: pre-oxidation with ozone, pH adjustment, activated carbon adsorption, coagulation/flocculation, sedimentation, filtration with sand filters, pH correction and disinfection with chlorine. This WTP is composed of two independent treatment lines, each one with the capacity to produce 120,000 m^3^/day. In WTP_D, surface water undergoes the following treatment processes: pre-oxidation with chlorine, remineralization and correction of aggressiveness, coagulation, filtration with sand filters, pH correction and disinfection with chlorine. This WTP has two independent treatment lines with the capacity to produce 500,000 m^3^/day (line 1) and 125,000 m^3^/day (line 2). These two WTPs provide water for more than three million inhabitants [37].

### 2.2. Water Collection and Primary Concentration

The sampled water analyzed was collected and processed (concentrated) in 2019, between January and December. Sampling sites, dates and volumes of sampled water are indicated in Table 1. Large starting volumes of water (Table 1) were concentrated using Nanoceram^R^ PAC-AG electropositive filters (Argonite; Sanford, FL, USA) set up in housing chambers [38,39,40]. The housings with the filters immersed in water (500 mL) were transported refrigerated to the laboratory, as soon as possible and processed within 72 h.

### 2.3. Elution and Secondary Concentration

The procedure was carried out according to EPA Method 1615 (EPA/600/R-10/181) with some modifications [41]. Before eluting the filter, 10 µL of *Mengo* virus solution (process control virus) with 10^5^ copies/µL (bioMérieux; Marcy-l’Etoile, France) was added to the water inside the housing with the filter [42,43]; then, this water was passed through the filter to be discarded. Following this procedure, the Nanoceram^R^ PAC-AG filters (Argonite; Sanford, FL, USA) were eluted with 1 L of 3% beef extract (BD Bioscience; Franklin Lakes, NJ, USA). The resulting eluted solution was subjected to an organic flocculation process with pH adjustment to 3.5, followed by centrifugation at 2500× *g*, 4 °C for 15 min The pellet was resuspended in sodium phosphate pH 7.0–7.5 and, after adjusting the pH to 9.0, a new centrifugation was performed at 5000× *g*, 4 °C for 10 min. The resulting supernatant was transferred to a new tube and the pH adjusted to 7.0–7.5. Samples were filtered through Acrodisc Syringe filters (PALL Corporation; Ann Arbor, MI, USA) with a pore size of 0.22 μm and the resulting volume (35–40 mL) was divided into three parts: 20 mL for RNA extraction and further evaluation by RT-qPCR, 10 mL for inoculation into cell cultures and 5 to 10 mL for storage. The samples were kept at −70 °C until use.

### 2.4. Tertiary Concentration and Nucleic Acid Extraction 

Samples reserved for RT-qPCR analysis (20 mL) were thawed and applied to Vivaspin^R^ concentrators (Sartorius; Goettingen, Germany) that were centrifuged at 8000× *g* and 4 °C for several hours including, in the final, two wash steps with 1 mL of 1 M sodium phosphate (pH 7.0–7.5), until the sample volume was less than 1 mL. The final concentrate was transferred from the Vivaspin^R^ concentrator to a 1.5 mL microtube. All final concentrates were subjected to RNA extraction and purification with the QIAamp viral RNA Mini kit (Qiagen; Hilden, Germany) [44,45] according to the manufacturer’s instructions.

### 2.5. Detection and Quantification of Viral Genomes

RT-qPCR amplifications were performed in a StepOnePlus thermocycler (Applied Biosystems; Foster City, CA, USA), in reaction mixtures of 25 µL containing 5 µL of extracted RNA. Each template RNA was assayed in duplicate and negative controls without nucleic acid as well as positive controls were introduced in each run. HEV and *Mengo* virus were assayed with a CeeramTools Hepatitis E kit (bioMérieux; Marcy-l’Etoile, France) and a *Mengo* virus Extraction Control kit (bioMérieux; Marcy-l’Etoile, France), respectively. HEV amplification conditions were reverse transcription at 45 °C for 10 min, enzyme activation at 95 °C for 10 min, followed by 40 cycles of amplification with denaturation at 95 °C for 15 s and data collection at 60 °C for 45 s. *Mengo* virus amplification conditions were reverse transcription at 45 °C for 10 min, enzyme activation at 95 °C for 10 min, followed by 45 cycles of amplification with denaturation at 95 °C for 15 s and data collection at 60 °C for 45 s. Quantification of HEV was estimated by standard curves, with five points constructed with serial dilutions (1:10) of control HEV RNA (CeeramTools Hepatitis E Standard kit; bioMérieux; Marcy-l’Etoile, France). *Mengo* virus quantification was performed with a four-point quantitation curve (0.1%, 1%, 10%, and 100%), prepared with RNA extracted from 10 µL of *Mengo* virus solution (10^5^ copies) with the QIAamp viral RNA Mini kit (Qiagen; Hilden, Germany). Only the results that met the quality criteria established by the mentioned kits were considered. Values given were the average of the results obtained in two independent RT-qPCR reactions. Initial results, expressed in genomic copies per five microliters of RNA (gc/5µL) in the reaction mixture, were converted in number of genomic copies per liter (gc/L) of sampled water, based on the data presented in Table 1. For *Mengo* virus, the results were expressed in percentage (%) of genomic copies (according to the quantitation curve).

### 2.6. Infectivity Assays

Concentrated water samples reserved for assays of infectivity were thawed and maintained at 4 °C, until inoculation into Vero E6 cultures (Vero C 1008, ATCC CRL–1586) [46,47]. Cells were grown at 37 °C in 25 cm^2^ flasks (T25) in the following culture medium (culture medium, FBS10): CO_2_-Independent Medium (Gibco, Thermo Fisher Scientific; Waltham, MA, USA) with additional 10% fetal bovine serum (FBS) (Gibco, Thermo Fisher Scientific; Waltham, MA, USA), 2 mM GlutaMAX (Gibco, Thermo Fisher Scientific; Waltham, MA, USA), and 0.5 mg/mL gentamicin (Gibco, Thermo Fisher Scientific; Waltham, MA, USA). Cells were routinely sub-cultured at confluency, by action of TrypLE Express cell dissociation reagent (Thermo Fisher Scientific; Waltham, MA, USA).

Cell inoculations with water samples were carried out in identical culture conditions, except that the concentration of FBS was 2% instead. The original culture medium was discarded from each T25 with a sub-confluent cell culture, prior to its inoculation with 1 mL of water sample diluted 1:2 in culture medium, FBS2. After 3 h of incubation with gentle agitation, 4 mL of FBS2 was added and incubations occurred until a cytopathic effect (CPE) was observed or for a maximum period of 15 days. Cultures were observed daily and when longer incubations were needed, there was a replacement of the culture medium every 5 days, keeping the previous medium refrigerated. Finally, cells (in suspension or manually detached) and culture medium were frozen/thawed (-70 °C/37 °C) twice and centrifuged at 3000× *g* for 5 min. Supernatants with the first-passage (P1) intra and extracellular putative viral particles (pVPs) were used in a new round of inoculations, performed as described above, in order to produce pVPs from a second passage (P2). However, P2 pVPs differ from P1 pVPs because at the end of the incubation period, the culture medium with cells was directly subjected to centrifugation to recover the supernatant, mostly consisting of extracellular pVPs. The P2 pVPs were subjected to a 6 h centrifugation at 17,000× *g* and 4 °C and the pellets were suspended in 140 µL of phosphate-buffered saline (PBS) in order to be processed for RNA extraction and purification, as indicated in 2.4. RT-qPCR targeting HEV RNA was performed as described in 2.5.

### 2.7. Statistical Analyses

All statistical analyses were conducted using Microsoft Excel 2017 (Microsoft Inc., Redmond, WS, USA) and IBM SPSS Statistics (SPSS Inc., Chicago, IL, USA). The Kolmogorov–Smirnov test was applied to assess the normality of the data within each data set; a *p*-value greater than 0.05 was indicative of a normal distribution. Once the normality was confirmed, the Student’s *t*-test could be used to compare the data. Differences were considered significant for a *p*-value less than 0.05.

## 3. Results

### 3.1. RT-qPCR HEV RNA Detection and Quantification in Water Samples

#### 3.1.1. HEV RNA in Surface Water Samples

**Figure 2:**
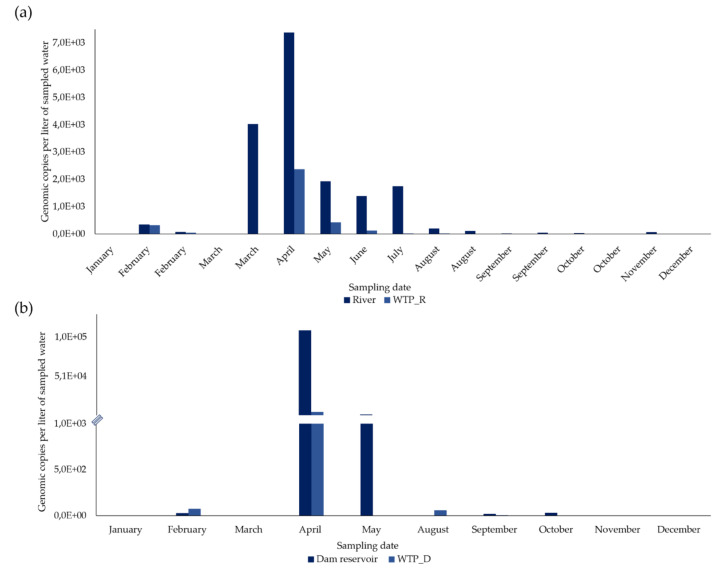
Variation in the concentration of hepatitis E virus (HEV) RNA detected in concentrated water sampled in four sampling sites during 2019. (**a**) River and WTP_R (*n* = 34). (**b**) Dam reservoir and WTP_D (*n* = 20). RT-qPCR results (average values from two independent reactions), in gc/L, indicate estimated genomic copies per liter of sampled water (based on data from Table 1).

**Figure 3:**
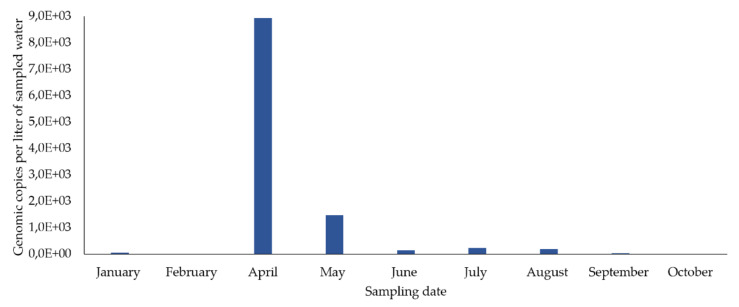
Variation in the concentration of HEV RNA detected in concentrated drinking water from the sampling point in the distribution network during 2019 (*n* = 9). RT-qPCR results (average values of two independent reactions), in gc/L, indicate estimated genomic copies per liter of sampled water (based on data from Table 1).

From the 27 concentrated samples from surface water, HEV RNA was detected and quantified in 21 (77.8%) (Figure 2 and Figure 3).

In the river, in February, March, August, September, and October, two sampling campaigns were carried out (in the first and second half of each month) and, of the 17 concentrated samples, 15 (88.2%) were positive for HEV RNA (Figure 2a). HEV RNA was detected in 11 of the 12 months of campaign, in concentrations fluctuating between 0 and 7383.2 gc/L, with a maximum in April (7383.2 gc/L) and a very sharp decrease from August to December (Figure 2a).

In the 10 concentrated surface water samples from the dam reservoir, HEV RNA was detected in six (60%) (Figure 2b). HEV RNA was detected and quantified in concentrations ranging between 0 and 10,9687.5 gc/L (the highest concentration, which was in April), with a concentration lower than 2411.9 gc/L in the remaining months (Figure 2b).

#### 3.1.2. HEV RNA in Drinking Water Samples

From the 36 samples of concentrated drinking water, HEV RNA was detected and quantified in 24 (66.7%) (Figure 2 and Figure 3).

In WTP_R, two sampling campaigns were carried out in February, March, August, September, and October (in the first and second half of each month). HEV RNA was detected in 11 out of 17 concentrated samples (64.7%) (Figure 2a), presenting its highest concentration (2,379.3 gc/L) in April, which then declined sharply to 0. It was not detected in January, March (first and second half), August and October (second half in both months), and December.

In the 10 concentrated drinking water samples from WTP_D, HEV RNA was detected in five (50%), corresponding to five months (Figure 2b) and was not detected in January, March, May, November, and December. From 75.151 gc/L detected in February and a maximum concentration of 5617.1 gc/L reached in April, HEV RNA concentrations significantly decreased, presenting values between 58.725 gc/L to no detection, from May to December. 

In the concentrated samples of drinking water from the sampling point in the distribution network, HEV RNA was detected in eight out of nine samples (88.9%) (eight months) (Figure 3), in concentrations fluctuating between 5645 and 8926.6 gc/L. The highest concentration occurred in April and then decreased until December. It was not detected in February.

### 3.2. Evaluation of the Efficacy of Water Treatment Plants (WTPs) in HEV RNA Elimination

#### 3.2.1. River vs WTP_R

In January and the first half of March, HEV RNA was not detected either in the surface water of the river or in its treated drinking water from WTP_R. Along the remaining months (15 sampling dates), HEV RNA was detected in the river, always in higher concentrations than in the WTP_R (9.8–100% reduction, as shown in Table 2). The lowest reduction values were obtained in February (9.8 and 37%, in the first and in the second half, respectively), while the highest values (100%) were obtained in the second half of March, August, October, and December; values higher than 87.6% were obtained in the first half of these months. High reduction values (>90%) were also obtained in four other situations (June, July, and in the first and second half of September), while intermediate values were obtained in the remaining comparisons (77.9 and 67.8%, in May and April, respectively). There were statistically significant differences in the concentrations of HEV RNA between samples from the river and from WPT_R, i.e., between non-treated and treated water samples (Student’s *t*-test, *p* < 0.05).

#### 3.2.2. Dam Reservoir vs WTP_D

In January, March, and December, HEV RNA was not detected either in surface water from the dam reservoir or in its treated drinking water from WTP_D, and in February and June, there was no reduction with the treatment. However, a reduction of 73.3% was observed in September and a reduction of over 94.9% in April, May, October, and November (Table 2). There were no statistically significant differences in the concentrations of HEV RNA between samples from the dam reservoir and from WTP_D, i.e., between non-treated and treated water samples (Student’s *t*-test, *p* > 0.05).

### 3.3. Recovery of the Process Control Virus (Mengo Virus) in the Water Samples Subjected to the Survey

**Figure 4:**
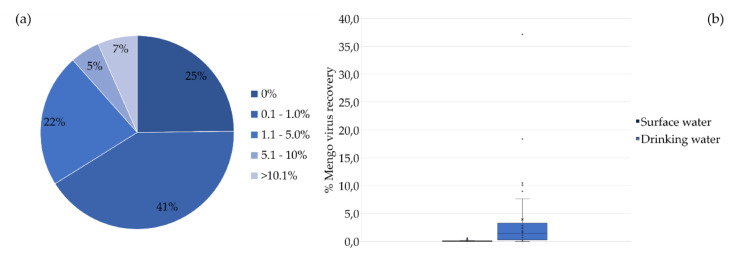
Recovery of the process control virus (%) in the water samples analyzed. (**a**) *Mengo* virus recovery in surface water and drinking water samples (*n* = 59). (**b**) Box plot generated with *Mengo* virus recoveries in samples from surface water and drinking water samples (*n* = 59); X represents the average recovery value.

*Mengo* virus recovery was evaluated by RT-qPCR in the 59 water samples subjected to this survey: 25 from surface water and 34 from drinking water. The percentages of recovery were low (0.1–5.0%) in most samples (63%), and it was not even detected in 25%. Recoveries higher than 5% were found in 12% of the samples (Figure 4a). The results, shown in Figure 4b, evidence much lower recoveries in surface water samples than in drinking water samples. There were statistically significant differences in the recovery of *Mengo* virus between surface water and drinking water (Student’s *t*-test, *p* < 0.05).

### 3.4. Potential Infectivity of the Water Samples

#### 3.4.1. Effect on Vero E6 Cultures and Production of Putative Viral Particles

Thirty-four concentrated samples from water sampled in 2019, between January and August and covering all sampling sites, were considered for infectivity assays in Vero E6 cultures. However, samples previously identified as negative for HEV RNA were not selected, except when the related sample (collected on the same date in the associated matrix) was positive. This was the case of the four HEV RNA negative samples, whose infectivity results are shown in Table 3 and Table 4.

Most cultures (19 in 32) did not develop CPEs during the incubation period (15 days). Nevertheless, putative viral particles (pVPs) from this first passage were collected and used to infect new cultures. From these, 17 developed CPEs within 2–6 days post-inoculation; pVPs from this second passage, with or without CPEs, were collected and subjected to RNA extraction.

#### 3.4.2. RT-qPCR Evaluation of Putative Infectious HEV Produced in Vero E6 Cultures

RNA extracted from pVPs produced as referred to above was subjected to RT-qPCR evaluation. From the samples evaluated, 18 were related samples from the river and WTP_R (eight from each) (Table 3), eight from the dam reservoir and WTP_D (four from each) (Table 4), and six were from the sampling point in the distribution network (Table 5). HEV infectivity was confirmed in samples from all matrixes (globally 25%): 3/13 (23.0%) from surface water were positive (two from the river and one from the dam reservoir: 22.2% and 25.0%, respectively) as well as 5/18 (27.7%) from drinking water (three from WTP_R, one from WTP_D and one from the sampling point in the distribution network: 33.3%, 25.0% and 16.6%, respectively) (Table 3, Table 4 and Table 5).

It was also possible to determine that 1) most positive samples for HEV infectivity had also tested positive for HEV RNA (exceptions were WTP_R from August and dam reservoir from June) and 2) positive samples for HEV infectivity were frequently found in related samples, i.e., in river/WTP_R and dam reservoir/WTP_D sampled on the same date (one exception was found in river/WTP_R from May, where only WTP_R was positive for infectious HEV) (Table 3, Table 4 and Table 5). Moreover, a relationship was not evidenced between the number of RNA copies detected in a water sample and its potential infectivity because, from the 11 samples presenting more than 1000 gc/L, only one (river from June) evidenced infectivity; values of gc/L between 0 and 428 had been found in all the others able to produce infectious HEV in Vero E6 cells.

## 4. Discussion

Although a preliminary work, this study followed a complex and complete approach in order to assess the presence of HEV, starting from high volumes of water and combining, in the same procedure, the possibility to detect viral RNA by RT-qPCR as well as evaluate infectivity. Other methods are limited to a maximum starting volume of five liters of water, as described in ISO 15216-1:2017 [42,43], but EPA Method 1615 of the United States Environmental Protection Agency [41], which supported this experimental protocol, is based on the collection of much larger volumes, making the results more reliable [40,48]. The use of cell cultures also overcame the limitation of evaluations based only on RT-qPCR. In fact, RT-qPCR has been increasingly used to detect enteric viruses in water and food samples, with high specificity/sensitivity and the possibility of obtaining results in less than four hours [3,48,49]. However, this methodology does not allow assessing the infectivity associated with the viral genomes detected in the reaction [3,25,40]. Beyond the confirmation of viral genomes, it is crucial in the evaluation of risks to public health in order to determine whether they correspond to viral particles with the ability to infect human cells as well [48,49,50,51]. Despite being expensive and time consuming, relying on cell cultures, it is the most used standard method for assessing the infectivity of viral particles, by observing cytopathic effects (CPEs) [3,6,52].

This one-year survey evaluated the presence of HEV in concentrated samples from two bodies of water (a river and a dam reservoir) and from the drinking water sampled on their water treatment plants (WTP_R and WTP_D, respectively) at the end of the treatment process. A mammal cell line (Vero E6) derived from African green monkey (Cercopithecus aethiops) kidney was used for the first time in order to assay the potential infectivity of water samples where HEV RNA had been detected by RT-qPCR. The rationale for the utilization of this cell line was its capability to replicate many different viruses [46,53], also taking into account that HEV has a large host range [12,15]. This approach effectively resulted in the detection of infectious HEV in several samples, by induction of CPEs in cultured cells with subsequent confirmation of HEV replication through RT-qPCR to RNA extracted from extracellular viral particles. Our results agree with a recent study [54] demonstrating that a wild-derived HEV strain replicated in Vero cells, the cell line from which Vero E6 was derived (46).

HEV was detected in concentrated samples from the two bodies of water, and in an infectious state in some of these samples. Comparing the two bodies of water, the river showed a greater number of positive samples for HEV RNA (88,2%) than the dam reservoir (60%). On the other hand, in both, the maximum concentration (>100,000 gc/L, in the dam reservoir) occurred in April (Spring in Portugal) and decreased until December. The maximum concentrations in April can be explained by the pattern of precipitation in Portugal, as described for Colombia [25]. In April 2019, rainfall was high, the fifth rainiest April since 2000 [55] and, associated with more precipitation, a greater runoff of possible contaminants from the surrounding areas may have disturbed these water bodies [56]. Many of the areas surrounding the sampling sites are places of agriculture and animal production [57], where manure from animal production activities is still used as fertilizer. It is well known that pigs are reservoirs of HEV, which is excreted in feces, and might thus be the possible origin of water contamination [2,58,59]. Concentrated surface water samples also presented infectious HEV in higher percentages (33.3%) in samples from the river than in samples from the dam reservoir (25%). Although the peak HEV RNA concentration was found in April, those samples did not produce infectious HEV in Vero E6 cells. Nevertheless, infectious HEV was detected in both bodies of water in June, when HEV RNA concentrations were high in the river (>1000 gc/L) and zero in the dam reservoir, evidencing inexistence of a direct association between the number of detected RNA copies and potential infectivity.

In drinking water, HEV RNA was also detected, although in a smaller number of concentrated water samples (64.7% in WTP_R, and 50.0% in WTP_D) than was observed in the bodies of water, as also found in Switzerland [9]. After analyzing the concentrated samples of drinking water from the two WTPs and from a point in the distribution network, there was an identical pattern: the peak HEV RNA concentration was detected in April, and subsequently decreased until the end of the year. These results are in complete accordance with what was previously described for the bodies of water that feed these WTPs and the point in the distribution network. Likewise, the highest HEV RNA concentrations were found in two related water matrixes: the dam reservoir and WTP_D. Out of the 18 concentrated drinking water samples selected for evaluation of HEV infectivity, 27.7% were positive. Drinking water from WTP_R presented the highest number of infectious samples (three), followed by WTP_D and the water from the point in the distribution network, both with only one infectious sample. Infectious HEV was detected in samples collected between May and August, after the peak of HEV RNA copies, as also observed in the bodies of water. No clear relationship was found between infectivity and number of HEV RNA copies detected per liter of sampled water.

Even though most of the results did not evidence contradictory aspects, a few should be discussed. One unexpected result was the infectivity of two samples (one from the dam reservoir and the other from the WTP_R) originally identified as negative for HEV RNA. This may be explained by eventual mishaps during the original RNA extraction procedures and emphasizes the relevance of evaluating results achieved by independent approaches. The detection of an infectious drinking water sample (WTP_R) was also unexpected, although the sampled water from its source (river) did not show infectivity. This may be explained by the large differences in the water volumes subjected to sampling: 1300 L in the WTP_R and 152 L in the river. It should be noted that the positive result of infectivity in WTP_R means that HEV was also present in the river to such an extent that infectivity remained after treatment.

The results showing the presence of infectious HEV in concentrated samples of drinking water evidence the need to further investigate eventual threats to human health. It is worth noting that Vero E6 cultures were inoculated with 0.5 mL of concentrated (40×, in average) drinking water samples, equivalent to approximately 17.5 L of the sampled water (1400 L, in average). Actually, while healthy individuals drink approximately two liters of water each day [60], this study, as referred above, was conducted with an average volume of 1400 L.

High values of genomic copies per liter found in several samples of natural water may also be attributed to the large volumes sampled. These large volumes were considered necessary to recover enough viruses to allow a reliable quantification based on the low levels of enteric viruses often reported for natural waters.

Low recovery percentages of the process control virus (*Mengo* virus) used for validation of the experimental procedure [61,62] were often found, as also occurred in Kyria da Silva et al. [63] and Teixeira et al. [43]. Recoveries were even lower in the untreated water, which may be related to the great number of particles and other interfering materials usually present [64]. These results suggest that *Mengo* virus was not a good recovery control despite the relevance that may be attributed to its presence at the end of the process for the validation of the eventual negative results of the viruses under detection.

Regarding the efficacy of the WTPs in reducing HEV, based only on genome copies, this was observed in both situations, but only with statistically significant differences in WTP_R. Differences in the water volumes sampled in the two types of matrix, as discussed above and evidenced in Table 1, may explain less marked differences. Differences in eliminating viruses may also be attributed to difficulties related to the manipulation of the sample during the various stages of filtration and concentration, including the characteristics of each water matrix. Moreover, the values obtained by RT-qPCR cannot be interpreted as absolute, since there are many steps in the experimental procedures that may lead to RNA degradation [48]. In any case, these results may indicate that the treatment at WTP_R, including pre-oxidation with ozone, adsorption with coal and disinfection with chlorine, may be more effective in the elimination of this virus than WTP_D, which uses neither ozone nor adsorption with coal [25,65,66]. Ozone may be crucial in the control of viruses in water, as found in other studies [67,68], concerning the elimination of enteric viruses and bacteriophages. However, more studies will be needed to confirm this statement.

Finally, climate change will certainly increase the frequency of pathogens in water systems worldwide, whether due to the occurrence of floods, sewage contamination or the scarcity of safe drinking water sources [3]. In this context and considering the results obtained in this study, monitoring the presence of HEV and other viruses in water supply and distribution systems is advisable. A similar approach should be conducted, increasing the sampling effort (number of samples and geographical regions during the entire year to allow the detection of possible seasonality patterns) and implementing the application of the quantitative microbial risk assessment (QMRA) [69].

## 5. Conclusions

HEV has is gaining an increasing presence in developed societies. As in many other countries in Europe and Asia, this virus also circulates in Portuguese waters. The results of this survey highlight the need for systematic monitoring of the presence of HEV and other emerging enteric viruses in surface and treated waters. It is also recommended to carry out studies targeting water treatment methods to better understand the influence of the various stages of the elimination/inactivation of these viruses in WTPs. 

## Figures and Tables

**Table 1:** Water surveyed from five sampling sites in 2019.

Sampling Site	Date	Sampled Volume (L)	
		Surface Water	Drinking Water
River and Water Treatment Plant R (WTP_R)	January	900	1200
	February, first half	690	1800
	February, second half	250	1300
	March, first half	220	1600
	March, second half	170	1500
	April	170	1350
	May	152	1300
	June	122	2000
	July	150	1500
	August, first half	150	1350
	August, second half	130	950
	September, first half	165	1200
	September, second half	220	1300
	October, first half	155	1400
	October, second half	130	1180
	November	130	1500
	December	230	1400
Dam reservoir and Water Treatment Plant D (WTP_D)	January	690	960
	February	710	620
	March	1500	3400
	April	550	1100
	May	2340	800
	June	530	1000
	September	450	900
	October	250	800
	November	665	945
	December	215	675
Point in the distribution network	January	-	1700
	February		1780
	April		1700
	May		1800
	June		1900
	July		1600
	August		1400
	September		1700
	October		1880

**Table 2:** Quantification of HEV RNA in concentrated samples from surface water sources and their associated water treatment plants, and evaluation of the treatment efficacy (reduction in RNA copies).

Date	HEV Concentration (gc/L)		Reduction (%) after Treatment	HEV Concentration (gc/L)		Reduction (%) after Treatment
	River	WTP_R		Dam Reservoir	WTP_D	
January	0	0	*	0	0	*
February, first half	355.5	320.8	9.8	-	-	-
February, second half	78.2	49.3	37.0	29.1	75.2	NR
March, first half	0	0	*	-	-	-
March, second half	4,029.1	0	100	0	0	*
April	7,383.1	2,379.3	67.8	109,687.5	5,617.1	94.9
May	1,936.5	428.0	77.9	2,412	0	100
June	1,394.9	126.0	91.0	0	58.7	NR
July	1,755.0	22.0	98.7	-	-	-
August, first half	206.5	24.2	88.3	-	-	-
August, second half	113.3	0	100	-	-	-
September, first half	23.3	1.9	91.9	-	-	-
September, second half	55.1	5.0	90.9	19.5	5.2	73.3
October, first half	36.3	4.5	87.6	-	-	-
October, second half	2.7	0	100	30.4	0.7	97.6
November	69.9	4.8	93.1	0.7	0	100
December	2.1	0	100	0	0	*

* Undetermined value or not calculated due to absence of detection; NR—no reduction with treatment; - no result, due to absence of sampling; gc/L: genomic copies per liter of sampled water, calculated with RT-qPCR results (average values of two independent reactions) and data from Table 1.

**Table 3:** Evaluation of related concentrated water samples (river and WTP_R) for the presence of HEV RNA and infectious particles.

Months	HEV RNA (gc/L)		HEV Reduction (%) after Treatment	HEV Infectivity (*)	
	River	WTP_R		River	WTP_R
February	355.5	320.8	9.8	Negative	Negative
February	78.2	49.3	37	Negative	Negative
March	4,029.1	0	100	Negative	Negative
April	7,383.1	2,379.3	67.8	Negative	Negative
May	1,936.5	428	77.9	Negative	Positive
June	1,394.9	126	91	Positive	Positive
July	1,755	22	98.7	Negative	Negative
August	206.5	24.2	88.3	Negative	Negative
August	113.3	0	100	Positive	Positive

*Based on RT-qPCR results, when RNA extracted from putative viruses produced in Vero E6 cultures were identified as HEV RNA; gc/L: genomic copies per liter of sampled water, calculated with RT-qPCR results (average values of two independent reactions) and data from Table 1.

**Table 4:** Evaluation of related concentrated water samples (dam reservoir and WTP_D) for the presence of HEV RNA and infectious particles.

Months	HEV RNA (gc/L)		HEV Reduction (%) after Treatment	HEV Infectivity (*)	
	Dam Reservoir	WTP_D		Dam Reservoir	WTP_D
February	29.1	75.2	NR	Negative	Negative
April	109,687.5	5,617.1	94.9	Negative	Negative
May	2,412	0	100	Negative	Negative
June	0	58.7	NR	Positive	Positive

*Based on RT-qPCR results, when RNA extracted from putative viruses produced in Vero E6 cultures were identified as HEV RNA; NR—no reduction with treatment; gc/L: genomic copies per liter of sampled water, calculated with RT-qPCR results (average values of two independent reactions) and data from Table 1.

**Table 5:** Evaluation of concentrated water samples from a sampling point in the distribution network, for the presence of HEV RNA and infectious particles.

Months	HEV RNA (gc/L)	HEV Infectivity (*)
January	46.9	Negative
April	8,926.6	Negative
May	1,473.5	Negative
June	133.3	Negative
July	221.4	Positive
August	186.6	Negative

* Evaluated by CPEs in Vero E6 cells, and RNA from virus produced in Vero E6 cells were found by RT-qPCR; gc/L: genomic copies per liter of sampled water, calculated with RT-qPCR results (average values of two independent reactions) and data from Table 1.
